# P-1010. Outpatient Management of Clostridioides difficile Infection in a Large Integrated Healthcare System

**DOI:** 10.1093/ofid/ofaf695.1207

**Published:** 2026-01-11

**Authors:** Jill Argotsinger, Jessica Miller, Victoria Gavaghan, Lynn Skelton, Catherine Passaretti, Robert J Citronberg

**Affiliations:** Advocate Lutheran General Hospital, Park Ridge, IL; Advocate Lutheran General Hospital, Park Ridge, IL; Advocate Health Care Midwest Region, Chicago, Illinois; Advocate Health, East Troy, Wisconsin; Advocate Health, East Troy, Wisconsin; Advocate Health, East Troy, Wisconsin

## Abstract

**Background:**

*Clostridioides difficile* infection (CDI) is known to be a major cause of infectious diarrhea in hospitalized patients. Recently, community-acquired CDI rates have been increasing. Accurate diagnosis of CDI can be challenging given the abundance of available laboratory tests with differing degrees of sensitivity and specificity. Per the Infectious Disease Society of America (IDSA) guideline, 2- or 3- step algorithms are recommended to increase the positive predictive value for diagnosis of CDI. Our health system performs a polymerase chain reaction (PCR), which when positive is reflexed to an enzyme immunoassay (EIA) for toxins A and B. For specimens that are PCR positive but EIA negative, an interpretation comment noting the results likely represent colonization or infection with low burden of disease is populated in the electronic health record. The purpose of this study was to evaluate management of PCR positive, EIA negative patients in the outpatient setting.

**Methods:**

This was a retrospective cohort study of adult and pediatric patients with PCR positive, EIA negative testing for *C. difficile* ordered during an outpatient visit within a large integrated health system and performed at a centralized laboratory from January 2023 to December 2023. Results were excluded if there was no documentation, order was sent as a test of cure, or if the assay failed.

**Results:**

A total of 456 patients with initial episode of PCR positive, EIA negative results were included in the study, with 406 patients receiving treatment for CDI and 50 patients receiving no treatment. Those treated for CDI were statistically more likely to have documented 3 or more watery stools in 24 hours (61.8% vs 48%, p< 0.01) with no difference in duration of symptoms. Risk factors for development of CDI had no impact on treatment including antibiotic exposure within the last 30 days, hospitalization within the last 90 days, proton pump inhibitor usage, household exposure or residence in a nursing home.

**Conclusion:**

Overtreatment of PCR positive, EIA negative patients was prevalent in the community setting. The only factor that may have influenced treatment was stool frequency. Traditional risk factors did not seem to influence treatment decisions.

**Disclosures:**

All Authors: No reported disclosures

